# Diversity of arterial cell and phenotypic heterogeneity induced by high-fat and high-cholesterol diet

**DOI:** 10.3389/fcell.2023.971091

**Published:** 2023-02-23

**Authors:** Jieqi Wen, Rongsong Ling, Ruiyue Chen, Siyan Zhang, Yarong Dai, Tingtao Zhang, Fanyu Guo, Qingxin Wang, Guixin Wang, Yizhou Jiang

**Affiliations:** Institute for Advanced Study, Shenzhen University, Shenzhen, China

**Keywords:** single-cell RNA sequencing (scRNAseq), western diet (WD), cellular heterogeneity, endothelial dysfunction (ED), atherosclerosis

## Abstract

Lipid metabolism disorder is the basis of atherosclerotic lesions, in which cholesterol and low-density lipoprotein (LDL) is the main factor involved with the atherosclerotic development. A high-fat and high-cholesterol diet can lead to this disorder in the human body, thus accelerating the process of disease. The development of single-cell RNA sequencing in recent years has opened the possibility to unbiasedly map cellular heterogeneity with high throughput and high resolution; alterations mediated by a high-fat and high-cholesterol diet at the single-cell transcriptomic level can be explored with this mean afterward. We assessed the aortic arch of 16-week old Apoe^−/−^ mice of two control groups (12 weeks of chow diet) and two HFD groups (12 weeks of high fat, high cholesterol diet) to process single-cell suspension and use single-cell RNA sequencing to anatomize the transcripts of 5,416 cells from the control group and 2,739 from the HFD group. Through unsupervised clustering, 14 cell types were divided and defined. Among these cells, the cellular heterogeneity exhibited in endothelial cells and immune cells is the most prominent. Subsequent screening delineated ten endothelial cell subsets with various function based on gene expression profiling. The distribution of endothelial cells and immune cells differs significantly between the control group *versus* the HFD one. The existence of pathways that inhibit atherosclerosis was found in both dysfunctional endothelial cells and foam cells. Our data provide a comprehensive transcriptional landscape of aortic arch cells and unravel the cellular heterogeneity brought by a high-fat and high-cholesterol diet. All these findings open new perspectives at the transcriptomic level to studying the pathology of atherosclerosis.

## 1 Introduction

As a chronic inflammatory disease, atherosclerosis is the major cause of myocardial and cerebral infarction and ischemia of the extremities, the underlying cause of about 50% of all deaths (Lusis, 2000). A disorder of lipid metabolism is the basis of atherosclerotic lesions, with cholesterol and low-density lipoprotein (LDL) acting as chief instigators (Lu and Daugherty, 2015). Several kinds of cells and cytokines are involved in the atherosclerotic progression.

Lesion of the diseased aorta originated from its intima. Minimally oxidized LDL diminishes nitric oxide to increase the permeability of endothelial layer and triggers endothelial cells to produce cytokines and chemokines, which helps monocytes pass through endothelial monolayer and convert into macrophages. Lipid accumulates here topically, and oxidation and modification of LDL happen under the presence of different enzymes and ROS. Macrophages then differentiate into foam cells with the help of highly oxidized LDL and gather. Under the regulation of cytokines, smooth muscle cells (SMCs) migrate into the inner lumen, and proliferate and secrete extracellular matrix (ECM) to promote the hyperplasia of fibrous cap and formation of plaque (Libby, 2000). Then macrophage and inflammatory T cells secrete various cytokines and enzymes to degrade matrix (Schönbeck et al., 2000), make the lesional plaque fragile, and induce calcification and precipitation. Finally the rupture of the plaque and the exposure of lipid core into blood recruit platelet initiate thrombosis. This blocks artery lumen and contributes to the ischemia or necrosis of organs or tissues supplied by the artery (Lusis, 2000).

It is clear that endothelial cells and macrophages play important roles in the development of atherosclerosis. Endothelial injury and repair are novel theories explaining pathogenesis of atherosclerosis (Mannarino and Pirro, 2008). Endothelial cells (ECs) are involved in a great range of homeostatic functions (Durand and Gutterman, 2013) through anti-coagulant, antithrombotic, and anti-inflammatory activity. Normally, endothelial cells regulate vascular tone, cell adhesion, and SMC proliferation (Paone et al., 2019). However, these functions lapse when pathological conditions appear and cause endothelial dysfunction (Werner et al., 2006). Most atherosclerotic risk factors can activate endothelial cells to secretes chemokines, cytokines, adhesion molecules, and intracellular adhesion molecules, hence aggregate immune cells (Davignon and Ganz, 2004). Macrophages play a vital role in the initiation of atherosclerosis and growth of plaque, while continuous inflammation leads to its apoptosis. In the absence of effective exocytosis, the accumulation of cell debris and apoptosis promotes the formation of a necrotic core in atherosclerotic plaques.

By thoroughly assessing the transcriptional landscape of aortic cells from mice administered with different diets, a panorama of the differences between normal and diseased endothelial cells can be looked at with the help of single-cell RNA sequencing. Western diet-mediated changes in immune cells, especially macrophages in the aortic arch, were also a focus in this study.

## 2 Materials and methods

### 2.1 Mice

Our mice strain came from the Nanjing Model Animal Resource Information Platform. Apoe^−/−^ male C57BL/6 mice (16 weeks old) were used to establish control and experimental group. APOE is often produced in monocytes and macrophages (Curtiss et al., 2000) and plays a critical role in blood lipid metabolism (Chen et al., 2017) as ligands for receptors that clear chylomicron and VLDL residue (Meir and Leitersdorf, 2004). So when APOE is knocked out, total cholesterol in plasma increases (Maganto-Garcia, Tarrio, and Lichtman, 2012), and the effect is multiplied especially under a high-fat and high-cholesterol diet. Female mice secrete estrogen, which lowers the content of LDL in plasma and enhances endovascular blood coagulation (Aryan et al., 2020). For the experimental group, to accelerate the progression of atherosclerosis, the mice were fed with high-fat and high-cholesterol food for about 12 weeks after they had been weaned (4 weeks old); this group is referred to as the Western diet (HFD) group for short. (Formula of high fat, high cholesterol diet: 20% sucrose, 15% lard, 1.2% cholesterol, 0.2% sodium cholate, 10% casein, 0.6% calcium hydrogen phosphate, 0.4% stone powder, 0.4% premix and 52.2% basal feed.) Meanwhile, another group of mice, the control group, was administered with a chow diet. Mice were euthanized after 12 weeks of being administered different diets. Animal studies were performed in compliance with ethical guidelines and use of animals, and the experimental protocol was approved by the Shenzhen University Animal Care and Use Committee.

### 2.2 Aorta dissection and single-cell suspension

After being euthanized, mice were locally perfused with cold PBS to remove the peripheral blood remaining in the aorta. The aorta were then harvested and incubated using the enzyme mix in the Lung Dissociated Kit (Cat# 130-095-927, Miltenyi Biotec). The aortic arch was cut into pieces before being immersed with the enzyme mix, and then the reaction was performed at 37°C for 40 min on a rotator. The product was then filtered through a 70 um strainer to remove the extra tissue, and the strainer was washed three times with Dulbecco’s modified Eagle’s medium (DMEM). The cell suspension was centrifuged for 5 min at 500×g, at 25°C. Pour off the supernatants and resuspend cells with DMEM again to obtain the final suspension.

### 2.3 scRNAseq

**FIGURE 1:**
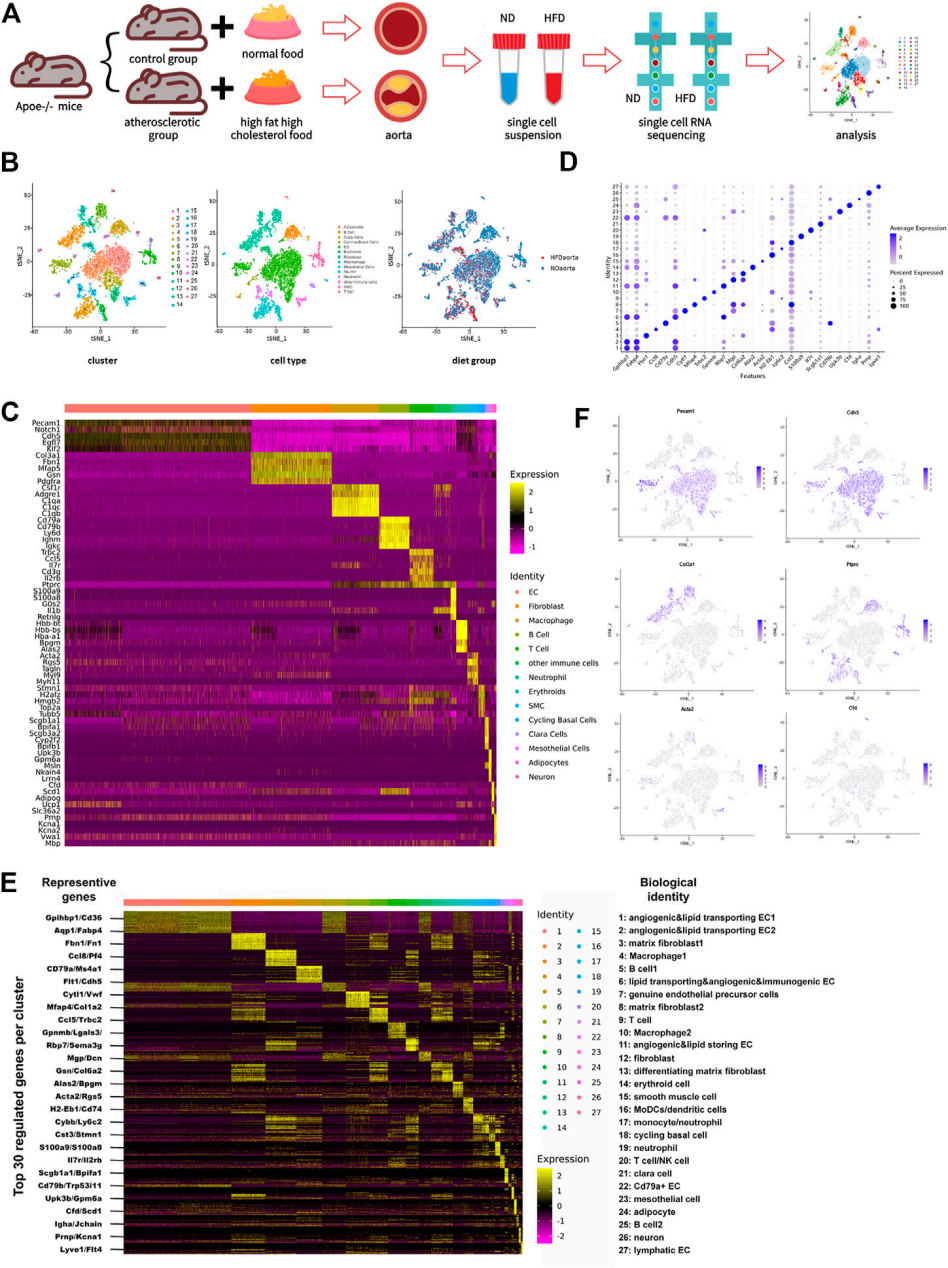
Transcriptome map of aortic cells extracted from both non-diseased and atherosclerotic Apoe^−/−^ mice. **(A)** Schematic diagram of the experimental flow. **(B)** t-Stochastic neighbor embedding (t-SNE) representation of gene expression information in single cells from control (ND) group and Western diet (HFD) group. Colors denote different clusters (left), cell types (middle), and groups (right). **(C)** Heatmap of five representative genes for each aortic cell type. **(D)** Dot plot demonstrates distinct marker of each cluster. Size of dot corresponds to proportion of cells expressing each transcript, and dot color corresponds to expression level of transcript. **(E)** Heatmap showing the top 30 upregulated genes (ordered by decreasing P value) in each cluster, with representative genes selected for biological identification of each cluster marked on the left and biological identification on the right. The color from yellow to purple symbolizes the expression levels from high to low. (Scale: log2 fold change) **(F)** Gene expression patterns projected onto t-SNE plot profile expression of six significant cell markers. Endothelial cell: Pecam1 and Cdh5; Fibroblast: Col3a1; Immune cell: Ptprc; Smooth muscle cell: Acta2; Adipocyte: Cfd.

Single-cell suspension of the two aortas from each group was pooled together as one sample. Single-cell RNA sequencing, library construction, and quality control were executed using the illumina-HiSeq3000 platform by Genergy Bio-technology (Shanghai) Co., Ltd. Dying cells with mitochondrial RNA above 30% and cells lacking information with UMI<200 were eliminated during the quality control, and 8,155 dissociated cells, with 5,416 cells from the control group and 2,739 from the HFD one, were filtered out thereafter. The median reads per cell were 3,955 for the control group and 3,421 for the HFD group, and transcripts detected per cell in the two groups were 1,364 and 1,069 respectively. (Figure 1A).

### 2.4 Data processing and visualization

The Cell Ranger Single-cell Software Suite was used to demultiplex the experimental data; Illumina’s bcl2fastq was wrapped around by using the mkfastq command.

The calculations based on UMI-tools were used to control quality of RNA sequencing. Sample libraries balancing was carried out for the number of estimated reads per cell and then ran on the illumine-HiSeq300.

Based on the 10x Genomics documentation (https://support.10xgenomics.com/single-cell-gene-expression/software/pipelines/latest/what-is-cellranger), demultiplexing, alignment filtering, barcode counting, UMI counting, and gene expression estimation were performed by Cell Ranger software on each sample. The LogNormalize algorithm worked in normalization. To compare experimental groups with normalized sequencing-depth and expression data, the IntegrateData (Seurat) was used to aggregate the gene expression estimates from each sample. Seurat (version 3.2.0) and R (version 4.0.0) package were used in downstream analysis. Cells with less than 200 genes were detected, and more than 30% of the mitochondrial gene count were filtered out as low quality or dying cells. Dimension reduction was then performed on normalized and logarithmized data by three stages of analysis, including the selection of variable genes, principal component analysis (nPCs = 50), and t-Distributed Stochastic Neighbor Embedding (t-SNE) with RunTSNE:dims = 1–30. Then the cell clustering was performed using the original Louvain algorithm (resolution = 0.9). We used the FindAllMarkers function to perform Wilcoxon rank-sum test based on the normalized data to identify gene markers in each cluster.

The Seurat package FindAllMarkers was used to analyze the differentially expressed genes (DEGs) between the two groups. The cowplot (version 1.1.1) and ggplot2 (version 3.3.5) were used for graphing. Sorting of endothelial cells were then performed with canonical markers Pecam1 and Cdh5 based on the estimated amount of EC in the total sample with resolution 0.4.

### 2.5 Serum collection

The mice to be sampled were individually isolated and kept in fasting conditions for 6 h, blood was collected below the jaw and stored in tubes without endotoxin. The samples were kept at 37°C for 1, 2 h to solidify the blood; blood was then left to clot overnight at 4°C. Afterwards, the serum was naturally precipitated, and centrifugation was performed for 10 min at 3,000 r/min at 4°C. Samples should be stored at −80°C if not used immediately.

### 2.6 Detection of CHO and TG in serum

The concentration of cholesterol and triglyceride were tested in serum with the Cholesterol Kit (CHOD-PAP Method) (Cat# 020080, Biosino) and Triglyceride Kit (GPO-PAP Method) (Cat# F001-2, Biosino). Prepare work solution. Mix work solution and cholesterol/triglyceride calibrator in different ratio to prepare cholesterol/triglyceride with different step concentrations. Mix work solution and sample in 100:1. The measured the concentration of cholesterol/triglyceride in serum using spectrophotometric method.

### 2.7 Statistical analysis

The statistical data of differentially expressed genes (DEGs) were calculated by Wilcoxon rank-sum test algorithm, and the threshold value of *p*-value was 0.05. The error bars in the bar graph represent standard error of mean (SEM). Two-tailed unpaired *t*-test and two-tailed Mann-Whitney test were used for statistical analysis unless stated otherwise. Statistical analysis was performed using GraphPad Prism version 8.0.2 or R version 3.2.0.

### 2.8 Single-cell suspension and flow cytometry

To prepare aortic cell suspension, fresh descending aorta and aortic root fragments, harvested from Apoe−/− male C57BL/6 mice (18 weeks old) fed with a Western diet and normal diet, three in each group, were incubated by an enzyme mix with 0.2 mg/mL Liberase (Roche, 5,401,054,001) and 2 U/mL Elastase (Sigma-Aldrich, E1250), with HBSS as the solvent. Digestion was done by rotating at 37°C in an oven for an hour. The product was filtered through the 35 um strainer and washed with HBSS. Cells were collected by centrifugation at room temperature, 500 xg for 5 min. The supernatant was discarded and the cells resuspended with staining buffer (3% BSA and 1%NaN in PBS).

Each group of cell suspension mentioned above was divided into four parts for incubation with the following four antibodies.

**Table:** 

Antibody	Preconjugated	Supplier	Cat No	Dilution
Anti-Vcam1	to FITC	abcam	ab33858	1:200
Anti-Cd36	to APC	abcam	ab82405	1:200
Anti-Pecam1	to FITC	ThermoFisher	11–0,311–82	1:200
Anti-Cdh5	to APC	ThermoFisher	11–0,311–82	1:200

Incubation was performed for 1 h at 4°C in darkness. Separate isotype controls for each antibody were also prepared. Secondary antibodies labeled with fluorescent dye were diluted with 3% BSA and used to resuspend cells at room temperature for 30 min in darkness.

Cells were washed with PBS by centrifugation at 400 *g* for 5 min twice. Finally, cells were resuspended with cold staining buffer (3% BSA and 1%NaN in PBS), the cell number was counted, and flow cytometry was performed. Cells were sorted by flow cytometry (CytoFLEX, Beckman Coulter) and analyzed with flow cytometer (CytoFLEX, Beckman Coulter) (version 2.0).

## 3 Results

### 3.1 Cell map of whole aortic arch

Whole-cell map of the aorta arch mapped by single-cell sequencing techniques has often been mentioned in the previous studies (Kalluri et al., 2019; Zhao et al., 2021). This study provides a more comprehensive map of the transcriptional information of all aortic cells. Cells from control and HFD group were pooled together and distinguished into 27 cell clusters corresponding to 10 different cell types through unsupervised clustering done using Seurat and then were visualized by the dimension reduction *via* t-stochastic neighbor embedding (Figure 1B). To define the identity of each cell cluster, we performed differential expression analysis between each cluster and assigned a specific identity to each cluster based on the established lineage-specific marker genes (Figure 1C). Some of these marker genes may were only evenly and slightly upregulated or just topically upregulated in certain clusters (Figure 1D), but this could not exclude the role of these marker genes in identifying cell clusters. (Kalluri et al., 2019).

The largest population of cells in this study is endothelial cells, accounting for 43.2% (Supplementary Figure 1A). Under such high-resolution sequencing, seven endothelial cell clusters were distinguished despite the similarity of partial expression profiles among the clusters. Different types of immune cells were distributed in nine clusters unevenly and account for 28.8%, the second largest population.

Occupying a proportion of 18.7%, fibroblasts identified by particularly positive expression of genes encoding fibronectin (like Col3a1, Fbn1, Mfap5, Gsn, and Pdgfra) were divided into four clusters. Traditional matrix fibroblasts in cluster three and eight were characterized by notable expression of Fn1 and Mfap4 respectively. Enhanced expression of Mgp and Dcn indicates that fibroblasts in cluster 12 were involved in composition of ECM and collagen, while differentiating matrix fibroblasts in cluster 13 were featured with high expression of Gsn and Col6a2, two genes involved in ECM processing. Except fibroblasts in cluster 12, which exist exclusively in the control group, all other fibroblast subsets exist in both groups.

Smooth muscle cells are always defined by their usual marker Acta2. This population also showed a rise of Tagln, Rgs5, Myh9, and Myl11. Other cell types include erythroid cell (Hbb-bt, Hbb-bs, Hba-a1, Bpgm, and Alas2), cycling basal cells (Stmn1, H2afz, Hmgb2, Top2a, and Tubb5), clara cells (Scgb1a1, Bpifa1, Scgb3a2, Cyp2f2, and Bpifb1), mesothelial cells (Upk3b, Gpm6a, Msln, Nkain4, and Lrrn4), adipocytes (Cfd, Scd1, Adipoq, Ucp1, and Slc36a2), and neurons (Prnp, Kcna1, Kcna2, Vwa1, and Mbp). Among these, cycling basal cells, clara cells, mesothelial cells, and adipocytes have not been identified in similar studies (Figures 1C–F; Supplementary Figures 1A–C).

### 3.2 Single-cell profile helps determine functionally distinct endothelial cell populations

**FIGURE 2:**
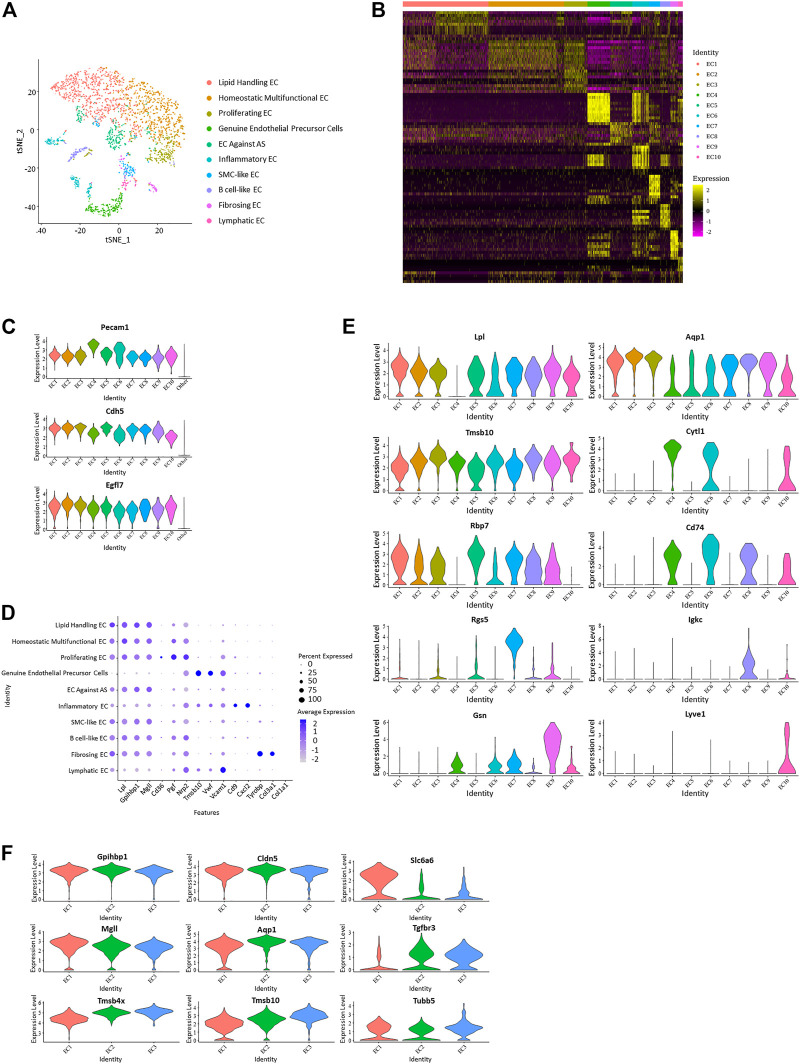
Differentiation of ten distinct vascular endothelial subpopulations. **(A)** t-Stochastic neighbor embedding representation of potential aortic endothelial cells extracted from total cell repertoire and separate them into 10 clusters. **(B)** Heatmap identifying top 10 upregulated genes of each endothelial subpopulation. **(C)** Violin plots of log-transformed gene expression of canonical endothelial markers distinguish these ten endothelial subpopulations from all other aortic cells. **(D)** Dot plot of functional markers showing the characteristics of each subpopulation. (Lipid handling: Lpl, Gpihbp1, Mgll, Cd36; Angiogenesis: Pgf, Nrp2, Tmsb10; Adhesion factor: Vwf, Vcam1, Cd9; Inflammation: Cxcl2, Tyrobp; Collagen forming: Col3a1, Col1a1) **(E)** Violin plots of log-transformed gene expression of selected markers demonstrating heterogeneity in each cluster. Violin plot y-axis demonstrates normalized transcript expression values. **(F)** Expression pattern of selected genes in endothelial subpopulation 1–3 showed heterogeneity and homogeneity between the three clusters. (Common: Gpihbp1, Cdh5; EC1 higher: Mgll, Slc6a6; EC2 higher: Aqp1; EC3 higher: Tgfbr3, Tmsb4x, Tmsb10, Tubb5).

Sorting of specific types of cells was performed based on single-cell gene profile and dependent on identity genes or specific conditions. 2,785 cells with positive expression of endothelial canonical markers Pecam1 and Cdh5 were selected as endothelial cell lines from the total cell repository, with 1786 from the control group and 999 from the HFD group. These cells were divided into ten subpopulations with specific identification (Figures 2A, B; Supplementary Figure 2A).

Positive expression of EC canonical markers Nos3 (Knowles et al., 2000), Ptprb, and Notch1 in these selected cells distinguish them from other vascular cells and support the lineage assignment as ECs. Genes encoding proteins worked in endothelial adhesion and angiogenesis, such as Pecam1 (Sauteur et al., 2014; Lim et al., 2019), Cdh5, and Egfl7 (Charpentier et al., 2013; Usuba et al., 2019), which also showed enrichment in these cells (Figure 2C; Supplementary Figure 2B). Genes with average log-fold enrichment >2 and *p*-value <0.01 are the first choice to be used in distinguishing between individual endothelial cell subpopulations.

Expression of different functional genes demonstrates the functional multiplicity of these endothelial cells. Markers that have a role in the transporting and metabolism of lipids Lpl (Lutz et al., 2001), Gpihbp1 (Allan et al., 2017), Mgll (Nomura et al., 2010), and Cd36 (Jabs et al., 2018; Gerbod-Giannone et al., 2019) were upregulated in almost all clusters except EC 4, 6, and 10. Cells in EC 4 and 10 worked in cell adhesion (Vwf and Vcam1), while cells in EC six were inflammatory (Cxcl2 and Tyrobp). Of note, the gene uniquely expressed in endothelial colony-forming cells, Cytl1, showed a particular rise in EC 4, suggesting it is a genuine endothelial precursor cell; because endothelial colony-forming cells was thought to be a late endothelial precursor cell with a strong angiogenic function distinct from classical endothelial cells (d’Audigier et al., 2018). This type of endothelial cell has not been mentioned in similar studies. It uniquely performed an undetectable expression of Lpl and presented a higher expression of Nos3. Lyvel, a validated lymphatic endothelial cell marker that plays a role in lymphatic reactions such as leukocyte trafficking and helping to clear acute inflammatory response after myocardial infarction (Okuda et al., 2012; Vieira et al., 2018; Jackson, 2019), showed an exclusive positive expression in EC 10.

In addition to the transportation or metabolism of lipids, other endothelial subpopulations also present with different characteristics. Genes related to angiogenesis and cell proliferation, such as Pgf, Nrp2, and Tmsb10, have higher expression levels in EC 3. In VECs, PPARγ plays a protective role by increasing nitric oxide bioavailability and preventing oxidative stress. As a PPARγ target gene, RBP7 (Hu et al., 2017; Fang and Sigmund, 2020) is enriched in VECs and upregulated in EC5 in this study, which suggests it as EC against AS. Smooth muscle cell marker Myl9 exhibited a high expression in SMC-like EC in EC 7. Rgs5, which is involved in the induction of endothelial apoptosis (Lovschall et al., 2007), also performed significant upregulation in EC 7. Encoding proteins involved in B cell proliferation, Igkc, Ighm, and Cd79a (Kedmi et al., 2011), present a specific rise in EC eight and are identified as B cell-like EC. Fibrosing EC was identified with greatly increased Gsn, Dcn, and genes that play a role in collagen forming (Col1a2, Col3a1, Col1a1) (Figures 2B, D, E; Supplementary Figure 2C).

Among different gene markers, there exists a notable negative correlation between Cd36 and Vcam1. All endothelial subpopulations, except EC 4, 6, and 10, expressed higher Cd36 and have a reduced expression of Vcam1, while the three excluded subpopulations showed inverse expression. Interestingly, Pecam1 and Cdh5 also performed similar negative correlation in these endothelial cells.

From previous analysis, EC 1–3 were found to lack genes with log-fold enrichment >1, indicating that these clusters lack significant features. However, by further analysis, we observed that the genes involved in cell proliferation and angiogenesis presented progressively elevated expression levels from EC1 to EC3, suggesting that they may be continuous phenotypic gradients rather than conventionally different subpopulations (Figure 2F). Analysis focused on the top 30 upregulated genes of endothelial subpopulations 1–3 further revealed that the expression profiles of some cells in cluster EC1 overlap with those of EC2, while almost all of the cells in cluster EC3 have the expression features of EC2 (Supplementary Figure 2D).

### 3.3 Comparison of transcriptional map between endothelial cells from two groups reveals diet-dependent genetic variance

**FIGURE 3:**
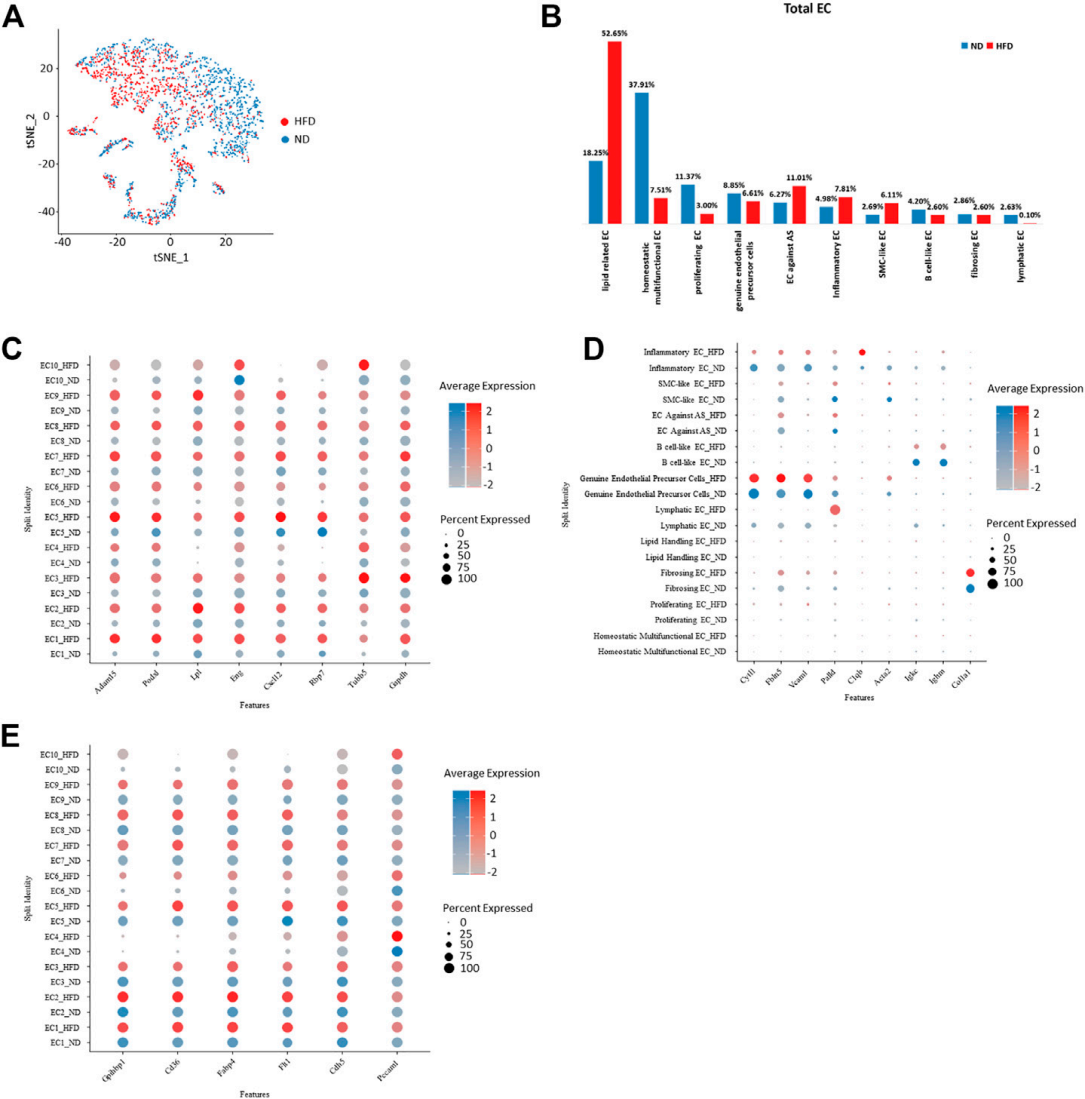
Homogeneity and heterogeneity between vascular endothelial cell of two origins uncover cellular variation respond to diet alteration. **(A)** t-Stochastic neighbor embedding representation of potential aortic endothelial cells extracted from total cell repertoire showing cellular origin in different colors. **(B)** Percentage of each endothelial population within total endothelial cells. Left: control (ND) group; Right: Western diet (HFD) group. **(C–E)** Transcriptional profiling of endothelial cells in ND *versus* HFD group notes diet-dependent genes with transcriptional upregulation in all HFD clusters. EC indicates endothelial cells **(C)** or diet dependent genes that are cluster specific **(D)** and conserved genes under regulation of diet. **(E)**.

Various endothelial subpopulations were distributed differently in the control group *versus* the HFD group. In total, the cells that exist preferentially in the HFD group indicated biological processes related with the development of mainly atherosclerosis. These subpopulations role in lipid clearance, utilization and storage, and exhibit property to against atherosclerosis but they also maintain role in inflammation and endothelial cell apoptosis. In contrast, those endothelial cells distributed mainly in the control group were involved in cell proliferating. Subpopulations EC1-3, which are assumed to have a developmental relationship, showed a distinct distribution between the two groups. EC1 has a biased distribution in the HFD group, while the other two were dominated by cells from the control group (Figures 3A, B).

The transcriptional profiles of endothelial cells in the two experimental groups can help to reveal which biological processes are regulated by diet. In addition, the biological processes not influenced by diet were listed. We have summarized all endothelial cell markers that were used in recent single-cell studies of atherosclerosis (Table 1) as a reference.

**TABLE 1:** Endothelial markers inferred in previous studies.

Gene name	Significance
HHcy	Involved in potentiates atherosclerosis mainly through endothelial injury and inflammatory activation Ma et al. (2022)
Mgp	Promoted ECs proliferation, migration, and tube formation Ni et al. (2022); ECM gene upregulated in HFD group, EndMT + EC and pro-inflammatory EC Zhao et al. (2021)
Nectin	Biasedly expressed in early stage of carotid atherosclerosis S. Li et al. (2021)
Egr1	Most enriched in regulatory regions in human vein and artery endothelial cells and has been predicted to act as a significant regulator of ECs under oscillatory shear stress S. Li et al. (2021), involved in cellular growth and development Huang et al. (2021)
Klf2	Significant regulator of anti-inflammatory response and maintenance of vascular integrity S. Li et al. (2021); Shear-sensitive gene F. Li et al. (2021); biasedly expressed in EndMT− ECs Zhao et al. (2021)
Klf4	Significant regulator of anti-inflammatory response and maintenance of vascular integrity S. Li et al. (2021); biasedly expressed in EndMT− ECs Zhao et al. (2021)
Gja4, Gja5, Arli5, Cd58	Expressed in EC related to coagulation cascade, viral myocarditis, and type I diabetes mellitus S. Li et al. (2021)
Sphk1	Involved in endothelial permeability S. Li et al. (2021)
Igfbp4, Plvap, Aqp1, Myc	Expressed in EC, mainly involved in ribosome-associated pathways, fluid shear stress in atherosclerosis, cancer proteoglycan, and leukocyte trans-endothelial migration S. Li et al. (2021)
Klk10	Unique EC marker (Williams et al., 2022) inhibits endothelial inflammation, endothelial barrier dysfunction, and reduces endothelial migration and tube formation (F. Li et al., 2021)
Vcam1	Inflammatory related marker Quiles-Jiménez et al. (2021) expressed in EC located in the lesser curvature of the aorta F. Li et al. (2021) and EndMT + cells Zhao et al. (2021). Expressed by activated endothelium, facilitates adhesion and transmigration of leukocytes, such as monocytes and T cells Depuydt et al. (2020)
VLDLR	Involved in uptake of lipoproteins, promoting foam cell formation under conditions of increased native or oxidized lipoproteins Liu et al. (2021)
Cd36	Receptor for oxidized low-density lipoprotein gene for lipid metabolism, biasedly expressed in lipid-handling EC F. Li et al. (2021); Zhao et al. (2021) (ref2)
ICAM-1	Shear-sensitive gene (Li et al., 2021), pro-inflammatory gene Sorokin et al. (2020); Zhao et al. (2021) (ref2,3) expressed in EndMT + ECs Zhao et al. (2021) (ref2)
BMP4	Hear-sensitive gene F. Li et al. (2021), involved in ECM organization Zhao et al. (2021)
Ang2, EZF/GKLF	Hear-sensitive genes F. Li et al. (2021)
Cavin2	EC marker, involved in maintenance and function of endothelial cells F Li et al. (2021)
Nos3	Antiatherosclerosis gene F. Li et al. (2021)
Clec3b, S100a4, Fmo2	Higher in early stage F. Li et al. (2021)
Cxcl2, Cxcl12, Jun, Tcf4	Higher in late stage F. Li et al. (2021)
Il6	Inflammatory gene Sorokin et al. (2020) higher in late stage F. Li et al. (2021)
Icam2	EC marker F. Li et al. (2021) involved in immunity and inflammation Huang et al. (2021)
Egfl7 Gu et al. (2019); F. Li et al. (2021), Vwf (F. Li et al. (2021); Zhao et al. (2021), Cytl1 F. Li et al. (2021), Cdh5 Garrido et al. (2021); Huang et al. (2021); Zhao et al. (2021), Cd34 Van Kuijk et al. (2019); Depuydt et al. (2020); Slenders et al. (2021), Pecam1 Gu et al. (2019); Van Kuijk et al. (2019); Depuydt et al. (2020); Zhao et al. (2021); F. Li et al. (2021); Huang et al. (2021); S. Li et al. (2021); Brandt et al. (2022); Burger et al. (2022); Ni et al. (2022); Pinheiro-de-Sousa et al. (2022)	Classical EC markers
Fn1	ECM gene biasedly expressed in HFD group, EndMT + EC and pro-inflammatory EC Zhao et al. (2021)
Tgfbr2, Bgn	ECM genes biasedly expressed in EndMT + EC Zhao et al. (2021)
Fgl2, Il7, Abca1, Eln	Biasedly expressed in EndMT + EC Zhao et al. (2021)
Ccl21a	Biasedly expressed in EndMT + EC Zhao et al. (2021) and lymphatic endothelial cell Cai et al. (2020)
Lpl, Gpihbp1	Biasedly expressed in lipid-handling EC Zhao et al. (2021)
Vim	Biasedly expressed in HFD group and pro-inflammatory EC Zhao et al. (2021)
Dcn	ECM gene (Zhao et al., 2021), involved in immunity and inflammation Huang et al. (2021), biasedly expressed in HFD group, EndMT + EC, and pro-inflammatory EC
Gapdh, Fabp4, Mgll	Involved in fatty acid metabolism, biasedly expressed in EndMT− EC Zhao et al. (2021)
Ctsb, Ctsz	Involved in ECM degradation Zhao et al. (2021)
Fabp5	Biasedly expressed in HFD group Zhao et al. (2021)
Cxcl6, Nfkbiz Zhao et al. (2021)	Pro-inflammatory gene biasedly expressed in EndMT + ECs Huang et al. (2021)
Lrg1, Ptprb, Acvrl1, Tmem100	Angiogenesis-related gene Huang et al. (2021)
Adamts1, Cd74, Cebpb, Ctla2a, Fcgrt, Kdm6b, Lcn2, Nfkbia, Sgk1	Involved in immunity and inflammation Huang et al. (2021)
Ecscr, Gpr56, Pcdh1, Tmsb10	Involved in cellular chemotaxis Huang et al. (2021)
Bmpr2, Ccdc85b, Fosb, Id3, Oaz1, Pfkbfb3, Tspan8	Involved in cellular growth and development Huang et al. (2021)
Cxcr3, Lyve1	Biasedly expressed in lymphatic endothelial cell Cai et al. (2020)

All genes enhanced in the HFD group are related to atherosclerosis. Some indicate the progression of disease, such as Adam15 (Langer et al., 2005; Oksala et al., 2009), Lpl (Dugi et al., 1997), and Gadph (Perrotta et al., 2014), which play a role in pathological neovascularization, lipid utilization and storage, and innate immunity, respectively. Some play roles in the dysfunction of endothelial cells, such as Podxl which functions as an anti-adhesive molecule (Shoji et al., 2018) and Eng which regulates endothelial cell shape changes in response to blood flow and is required for normal structure (McAllister et al., 1994; Vicen et al., 2019). Some are anti-atherosclerotic, such as Cxcl12, which plays a protective role after myocardial infarction (Hartmann et al., 2015), and Rbp7, which helps to increase nitric oxide bioavailability (Figure 3C).

Cluster-specific markers also performed variously in the two groups and further confirmed the effect of diet. Cytl1 and Vcam1, markers of genuine endothelial precursor cells, showed unchanged expression under different types of diet, as does the marker of fibrosing EC, Col1a1. Fbln5, which is reinduced in atherosclerotic lesions, showed a rise in the HFD group. Similarly, canonical B cell marker Igkc and Ighm showed a reduced expression under the regulation of the Western diet (Figure 3D). The cells in the experimental group lost a lot of primary endothelial cell functions, such as cell formation, adhesion, and contraction, but inflammatory responses and some lipid-related functions (markers including Gpihbp1, Cd36, and Fabp4) were still retained (Figure 3E).

### 3.4 Identification and diet-dependent variation of aortic immune cells under single-cell RNA sequencing

**FIGURE 4:**
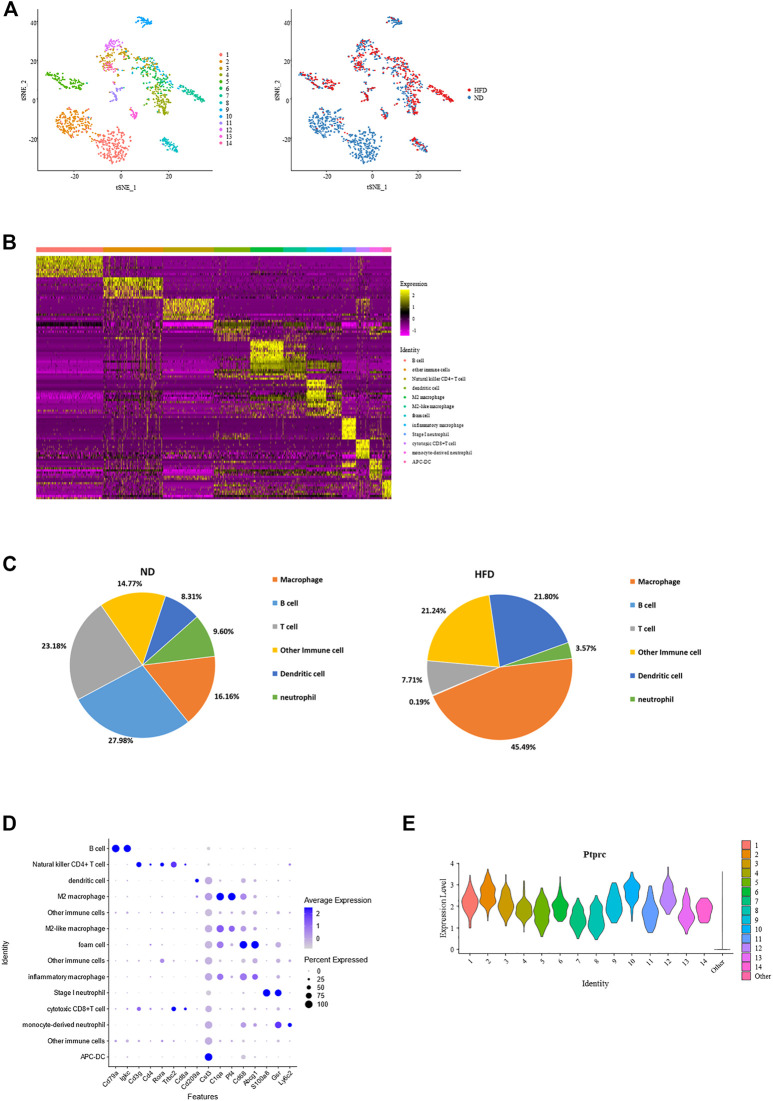
Identification of each immune cell cluster and their distribution reveal constitution of aortic immune cells and effect of diet. **(A)** T-Stochastic neighbor embedding (t-SNE) representation of gene expression information in aortic Ptptrc + immune cells. Cells from both control and Western diet group (right) are distinct into 14 clusters (left); CIBERSORT helps with their identification (middle). **(B)** Heatmap showing the top 10 upregulated genes in each cluster of immune cells, with biological identification of each cluster marked on the right. **(C)** Percentage of each immune cell type within total immune cells. Left: control (ND) group; Right: Western diet (HFD) group. **(D)** Dot plot showing the expression levels of representative marker genes for each immune cell type. **(E)** Violin plots of log-transformed gene expression of Ptprc distinguish these immune subpopulations from all other aortic cells.

Immune cells are another emphasis in this study, and we define Ptprc + cells as immune cells. 1,615 cells were filtered out from the total cell population, with 1,083 from the control group and 532 from the HFD group. These cells were reclustered into 14 subpopulations. Identification of these cells was done based on the transcriptional profiles with the application of CIBERSORT (Figures 4A, B; Supplementary Figure 3A).

Constitution of immune cells in the control group *versus* the HFD group reveals the great effect created by the Western diet. The most obvious heterogeneity brought by the Western diet was that B cells and T cells dominated the control group, accounting for 27.98% and 23.18% respectively, while macrophages had the largest proportion in HFD group, up to 45.49%. These 3 cell types dominate the immune cell. Another variance was the increase of dendritic cells and decrease of neutrophil (Figures 4A, C; Supplementary Figure 3A).

Macrophage had the largest proportion in all immune cells are were divided into four clusters, all showing a common expression of C1qa (De Couto et al., 2019). Pf4, an M2 macrophage marker, was expressed the highest in cluster4 and cluster6, while foam cell marker Cd68 and Abcg1 was enriched in cluster7. The proportion of B cell and T cell were similar. It should be noted that B cells (Cd79a, Cd79b, and Ly6d) existed exclusively in the control group. T cells (Trbc1, Trbc2 (Lefranc, 2014), Cd3d, and Cd3g) consisted of natural killer CD4^+^ T cells, Rora (Kästle et al., 2017), Il7r (Al-Mossawi et al., 2019), Icos (Niu et al., 2018) and Cd4, and cytotoxic CD8^+^ T cells, Cd8a and Cd8b1. The remainder of immune cell populations included two clusters of dendritic cells (DCs) with gene signatures Cd209a (cluster 3), Cst4 and Clec9a (cluster 14), two clusters of neutrophils (cluster 10: S100a8 and S100a9; cluster 10 and 12: Gsr (Yan et al., 2013); cluster 12: highly expressed Ly6c2, a marker of monocyte), and three other clusters of Ptprc + cells (Figures 4B, D; Supplementary Figure 3B). Regardless of type, all showed a high expression of Ptprc (Figure 4E).

### 3.5 Differentiation of macrophage and heterogeneity brought by wester diet

**FIGURE 5:**
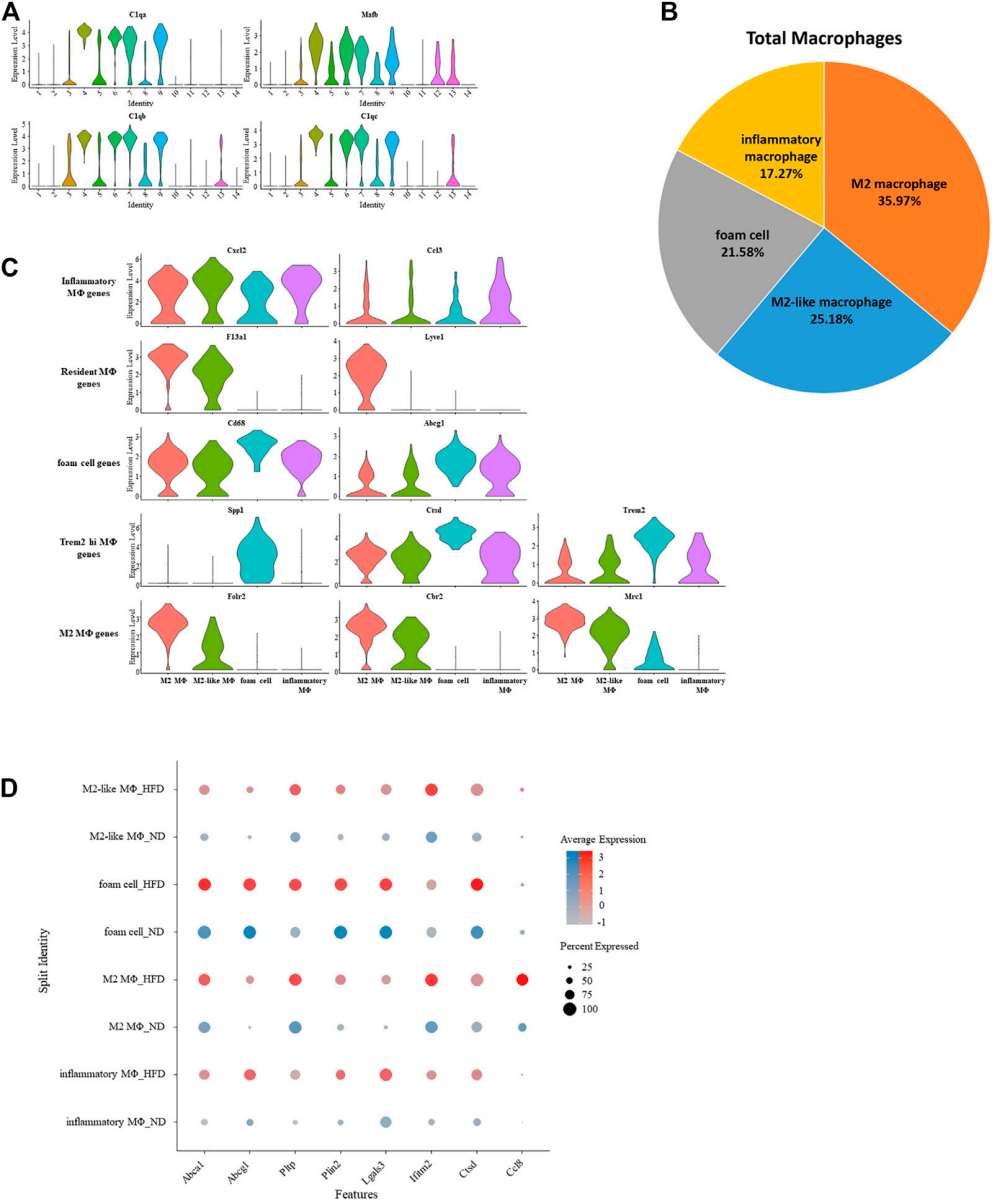
Gene expression signature of macrophage and its variation under western diet. **(A)** Violin plots of log-transformed gene expression of canonical macrophage markers in macrophages and all other aortic immune cells. **(B)** Percentage of each macrophage type. **(C)** Violin plots profile gene expression of markers for the five classes of macrophages in these selected macrophages. **(D)** Heterogeneity within macrophage of two origins demonstrated by dotplot.

As previously seen, four clusters were defined as macrophage. Common enrichment of C1qa (De Couto et al., 2019), C1qb (Giladi et al., 2018), C1qc (Zhao et al., 2020), and Mafb (Goudot et al., 2017) further confirmed the identity of macrophage (Figure 5A). The proportion of macrophage across all immune cells increased due to the appearance of foam cells according to the constitution of macrophage in the control group *versus* the HFD group (Supplementary Figure 4A). In the meantime, the proportion of M2 macrophage decreased. The remainder are M2-like macrophages and inflammatory macrophages (Figure 5B).

Single-cell differential expression pattern helps differentiate these four macrophage subsets from one another, and specific enrichment of these genes in each subset helps further determine their identification. Previous studies established a gene pool for various macrophages. Despite greater expression of M2 macrophage markers Folr2, Mrc1, and Cbr2, M2 macrophage also had characteristics of resident macrophage (F13a1 (Beckers et al., 2017) and Lyve1 (Ensan et al., 2016)). The expression profile of M2-like macrophages is similar to M2 macrophages, inferred by a transitional relationship between them. Meanwhile, due to the high expression of the monocyte marker Ccr2, we speculate that it is the progenitor of M2 macrophages. Foam cells were identified because the relative upregulation of Cd68 and Abcg1 can promotes cholesterol accumulation, which matters in the formation of foam cells (Cheng et al., 2016). These cells also have an exclusively positive expression of Spp1. Spp1 encodes osteopontin, which is a factor related to the severity of lesions. Another related factor is cathepsin, encoded by Ctsb, Ctsd, and Ctsz (Cochain et al., 2018), which was expressed higher in the foam cell. This was also the case with Trem2, a putative factor in Trem2-high macrophages except osteopontin and cathepsin. With significant upregulation of several chemokines (Ccl3, Ccl4, Cxcl1, Cxcl2, and Cxcl16) that play a role in inflammation, inflammatory macrophages were identified (Figure 5C; Supplementary Figure 4B).

The effect of diet on macrophages can also be seen from genetic regulation under the Western diet. These genes were involved in biological pathways such as cholesterol transporting (Abca1, Abcg1, and Pltp), development of adipose tissue (Plin2), inflammation (Lgals3 and Ccl8), and cell apoptosis (Ifitm2 and Ctsd) (Figure 5D).

### 3.6 Differential expression of Pecam1 and Cdh5 between different regions of the aorta

Pecam1 and Cdh5 are a pair of genes used to identify endothelial cells in this study. According to the scRNA sequencing data, an endothelial subpopulation, identified as genuine endothelial precursor cells specifically, was higher in Pecam1 and lower in Cdh5 compared to other endothelial subpopulations (Figure 1F). This subpopulation expressed Cytl1 exclusively (Figure 1D).

The Cytl1+ cells were found in three clusters in further analysis of sorted Pecam1+ Cdh5+ endothelial cells (Figure 2E), and retained the characteristics of high Pecam1 expression and low Cdh5 expression (Figure 2C). Upregulated Vcam1 expression level is another characteristic of these cells.

In the comparison of gene expression levels for endothelial cells isolated from a normal diet *versus* Western diet, Pecam1 and Cdh5 were listed in top 100 differentially expressed genes (DEGs), with Pecam1 lowered and Cdh5 enhanced in the Western diet group (Figure 3E).

**FIGURE 6:**
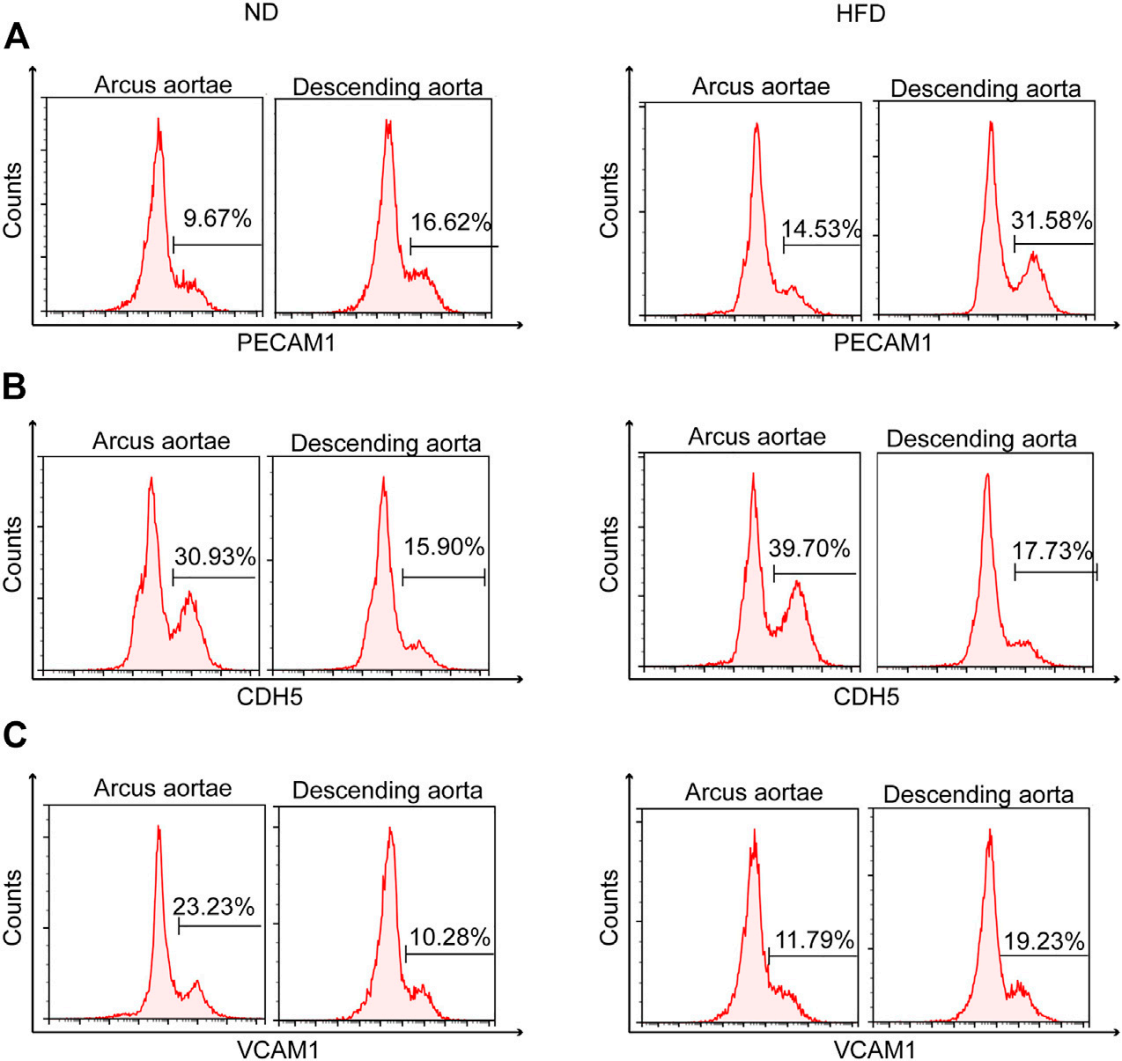
Identification of genetic heterogeneity *in situ* within endothelial cells. Flow cytometry of mouse aortic endothelial cells demonstrating heterogeneity of **(A)** Pecam1, **(B)** Cdh5, and **(C)** Vcam1 between greater (arcus aortae) and lesser (descending aorta) curvature of aorta, showing the influence of a Western diet.

Flow cytometry helped identify the distinct spatial location of high Pecam1 (Figure 6A) and low Cdh5 (Figure 6B) expression. Greater curvature of the aorta demonstrates less presence of high Pecam1-expressing cells and more presence of low Cdh5-expressing cells. The preference in location was found in both diet groups. Otherwise, the Western diet presented mild inhibition of the increase of low Cdh5-expressing cells.

Flow cytometry also helped identify the distinct spatial location of high Vcam1 expression (Figure 6C). High Vcam1-expressing cells showed a preference in descending aorta compared to the greater curvature of the aortic root under a high fat diet. A contrasting result was found under normal conditions.

## 4 Discussion

Known as the region with disturbed blood flow, the aortic arch is composed of different cell types and is relevant in the progression of cardiovascular disease and is the research object in this study. With well-established single-cell RNA sequencing, we characterized different types of cells in the aortic arch and mining gene expression changes that occur within these cells under the influence of diet. We differentiated 14 classes of cells from the total sample, including endothelial cells, fibroblasts, smooth muscle cells, mesothelial cells, adipocytes, neurons, and immune cell populations, which were distributed in 27 clusters. Notably, the identification of cycling basal cell, clara cell, mesothelial cell, and adipocyte was new. Heterogeneous gene expression pattern distinguishes 10 subpopulations of endothelial cells with distinct functions and reveals which biological processes may be affected or remain unchanged under a Western diet. This study identifies genuine endothelial precursor cells for the first time in such studies, complementing the transcriptional information blank of this cell. Another class of cells focused on in this study was immune cells, especially macrophages. Seven immune cell types were distributed in 14 immune cell clusters, and four clusters of macrophages expressed different characteristics. A genes expression map uncovered the multiplicity of aortic cells and demonstrated that the Western diet accelerates atherosclerotic development by regulating the role of the genes in various biological processes.

In the total aortic cell pool, the seven endothelial subpopulations were similar in the expression levels of Pecam1 and Cdh5. The cell adhesion molecules encoded by Pecam1 and Cdh5 are essential for leukocyte trans-endothelial migration (Dasgupta et al., 2009) and the maintenance of vascular lumen homeostasis (Lampugnani et al., 2010). Negative expression correlation between these two genes was observed in each endothelial subpopulation. A genuine endothelial precursor cell exhibited higher Pecam1 and lower Cdh5 gene expression levels compared to the other endothelial cell clusters.

Another gene pair showing similar negative correlation between endothelial precursor cells and other endothelial cells is Vcam1 and Cd36. Based on the conclusions of previous single-cell studies, Vcam1 and Cd36 were relatively biased to be lower and higher expressed in classic endothelial cells and endothelial cell roles in lipid treating. A classic endothelial cell tends to be distributed in descending aorta with less curvature, while other endothelial cells tend to be distributed in the arterial root with greater curvature (Kalluri et al., 2019). Genuine endothelial precursor cells exhibited the same expression pattern as the classic endothelial cells mentioned previously. According to the relationship between the two gene pairs, the endothelial precursor cells involved in this study can be speculated to be like classic endothelial cells, possibly located at sites with smaller curvature. Sites with less curvature often suffer attenuated lipid accumulation and lower atherosclerotic risk rates. The result of flow cytometry supports the speculation on the location of genuine endothelial precursor cells and demonstrates the influence of the Western diet on this cell type. Nos3, known for its promotion effects in nitric oxide production and vasodilation, was significantly upregulated in these precursor cells, further indicating the lower atherosclerotic risk rate within it (Lusis, 2000). Data in the STRING database showed a co-expression relationship between Cdh5, Pecam1, and Vcam1, while Cd36 was not involved. However, whether the supposed relevance among these proteins are reliable and how to localize them remains to be addressed.

Flow cytometry was not used in this study, instead only algorithm was used in the cell sorting. Endothelial cells sorted this way in the study showed a similar proportion of genuine endothelial precursor cells in both experimental groups. Except genuine endothelial precursor cells, inflammatory endothelial cells also had a higher expression of Pecam1 and Vcam1 as well as a lower expression of Cdh5 and Cd36. The two endothelial cell subpopulations existing in both the control and HFD group indicated that some precursor cells and inflammatory cells from advanced atherosclerosis were clustered together with cells in a normal condition. It can be speculated that the transcriptional profiles of these cells in the late disease and normal states are similar, but whether they locate in atherosclerotic plaque remains to be determined.

Distribution of EC1-3 and expression profile of them indicates great probability that endothelial develops from the state of EC3 into EC1 during the disease progression. During the process, endothelial went through dysfunction and lost the function of angiogenesis and cell proliferation.

The Western diet can accelerate the progression of atherosclerosis (Supplementary Figures 5A–D) as well as endothelial dysfunction. Genes that showed an increased expression indicated the character of dysfunctional endothelial cells. These dysfunctional endothelial cells lost cell adhesion function (Podxl), which is one of the key functions in normal endothelial cells. Meanwhile, based on certain responses, based on certain responses, they appeared to inhibit the further dysfunction of endothelial cells (Hu et al., 2017), for example, changing shape in response to blood flow in order to keep normal structure (Eng) or promoting the production of nitric oxide (Rbp7) (Fang and Sigmund, 2020). In accordance with previous findings, Cxcl2 is enriched in endothelial cells in advanced atherosclerosis and suppresses atherosclerosis after myocardial infarction (F. Li et al., 2021). As a key enzyme in triglyceride metabolism, Tubb5 plays an important role in removing lipids from the blood, as well as in lipid utilization and storage (Weinstock et al., 1995). The enrichment of these biological processes suggests that in most cases, feedback inhibitory pathways for atherosclerosis development are activated in dysfunctional endothelial cells, potentially limiting further plaque expansion by inhibiting lipid accumulation and regulating vasodilation in the lesion.

Both Fbln5 and Vcam1 were upregulated in genuine endothelial precursor cells of mice with advanced atherosclerosis administered with a Western diet, implying that it induced more leukocytes that migrated to sites of inflammation. At the same time, lipid-related functions in these cells were not as active as in other endothelial cells, suggesting their absence in proatherogenic biological processes such as cholesterol transport, lipid utilization, or storage.

The results showed that some immune cells were absent in the HFD group, such as B cells and CD4 + T cells, indicating that the Western diet may inhibit the differentiation of aortic immune cells. A recent study pointed out that B1a and B1b lymphocytes produce IgM to inactivate oxidation-specific epitopes on LDL and thereby protect against atherosclerosis (Pattarabanjird et al., 2022). Therefore, it can be speculated that B cells initiate protective mechanisms in atherosclerosis, while the Western diet reduces the number of B cells, thus aggravating atherosclerosis. In accordance with a recent study published in *Frontiers in Immunology*, which indicated that neutrophils and APC-like neutrophils were dominant in the blood of hyperlipidemic patients rather than healthy patients (Zhao et al., 2022), neutrophils and dedicated antigen-presenting dendritic cells (APC-DC) were found to be higher in the HFD group than in the control group. However, in this study, there was no more research into the role of APC-DC in atherosclerosis. This could be further explored in the future.

The proportion of macrophages observed in Ptprc + cells increases with disease progression, similar to the findings of other researchers (Galkina et al., 2006; Cochain et al., 2018). In contrast, the proportional increase in macrophages observed in this study was caused by foam cells rather than resident macrophages or inflammatory macrophages. These foam cells were specifically enriched with the validated markers of Trem2 high macrophage. It can be inferred that the transition from macrophages to foam cells occurs in the late stages of disease and the cell of origin may be highly expressing Trem2. During the progression of atherosclerosis, the production of foam cells is inseparable from the high oxidation of LDL. High oxidization assists LDL recognition by the scavenger receptor Cd68 and thus uptake by macrophages occurs rapidly. Clearly, the significant upregulation of Cd68 with the above principle could explain the emergence of foam cells in this study.

The role of Abca1, Abcg1, and Pltp in foam cells is particularly apparent in the late stage of atherosclerosis. They help with the efflux of cholesterol in macrophages and uptake into HDL, hence promote the conversion of LDL to HDL. This facilitation of HDL synthesis predicts an inhibited conversion of macrophages to foam cells and can be one of the manifestations of feedback inhibition in advanced atherosclerosis, which slows down the further development of disease.

In conclusion, transcriptional information of aortic cells, especially VEC, was mapped using single-cell RNA sequencing. Significantly, we identified 10 endothelial subpopulations and established candidate marker genes of each subpopulation. In addition, all endothelial candidate markers identified in previous research of atherosclerosis with single-cell RNA sequencing were confirmed in this study. Notably, by observing its transcriptional signature, we found genuine endothelial precursor cells, which had not been mentioned in previous studies. Compared with previous studies, the results of this study broaden the understanding of endothelial cells and further enrich existing information about different endothelial cell markers.

In addition, comparison of gene expression profiles and constitutions of cell between the health and atherosclerotic group can help in understanding the post-modification induced by a high-fat high-cholesterol diet in various cells, especially endothelial cells and immune cells. Understanding the pathways involved in these modifications may contribute to further exploration of pathogenesis of atherosclerotic lesions.

There are some limitations to this study, which may comprise further areas of research. Validation of several newly demonstrated genes or pathways has not yet been carried out. Traditional methods such as immunohistochemistry and western blot could be used to validate the distribution of these genes and pathways more accurately.

## Data Availability

The datasets presented in this study can be found in online repositories. The names of the repository/repositories and accession number(s) can be found below: https://www.ncbi.nlm.nih.gov/geo/, GSE206239.

## Abbreviations

DC, Dendritic cell; EC, Endothelial cell; ECM, Extracellular matrix; HDL, High-density lipoprotein; HFD, Western diet; LDL, Low-density lipoprotein; SMC, Smooth muscle cell; t-SNE, t-stochastic neighbor embedding; VEC, Vascular endothelial cell.
