# Supplementary material for: Diversity of arterial cell and phenotypic heterogeneity induced by high-fat and high-cholesterol diet
Source: Front Cell Dev Biol. 2023 Feb 23;11:971091. doi: 10.3389/fcell.2023.971091 (PMC9997679; doi:10.3389/fcell.2023.971091)
Supplement: Supplementary file 1 [file DataSheet1.PDF]

frontiers

Supplementary Material

1 Supplementary Figures

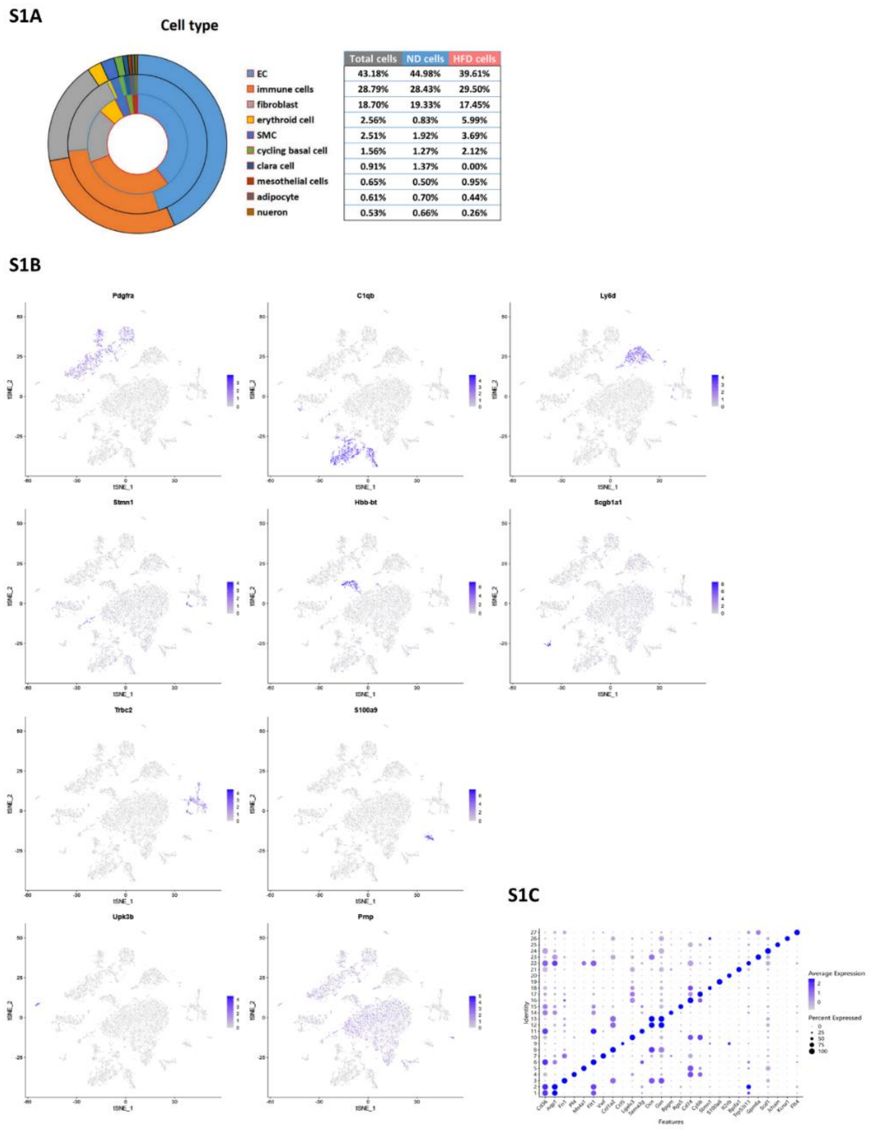

Supplementary Figure 1. (A) Proportion of each type of cell identified in the total cell repertoire, chow diet cell repertoire and western diet cell repertoire. **(B)** Gene expression patterns on tSNE plots reflect gene expression patterns of 10 additional cell markers for different cell type. **(C)** Expression pattern in dot plot demonstrate genes uniquely upregulated in each cluster from all aortic cells extracted from chow and western diet Apoe-/- mice. Dot size and dot color manifest the proportion and expression level of cells expressing each gene within the supposed group, respectively.

S2A

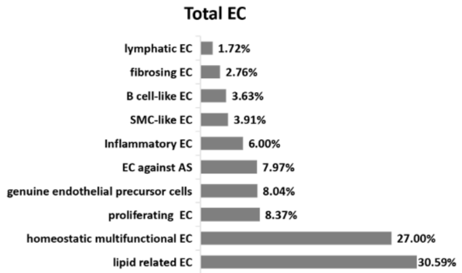

S2B

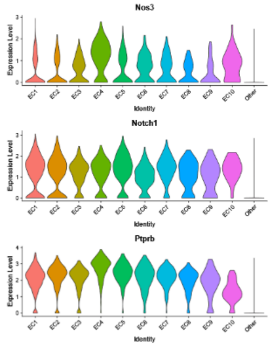

S2C

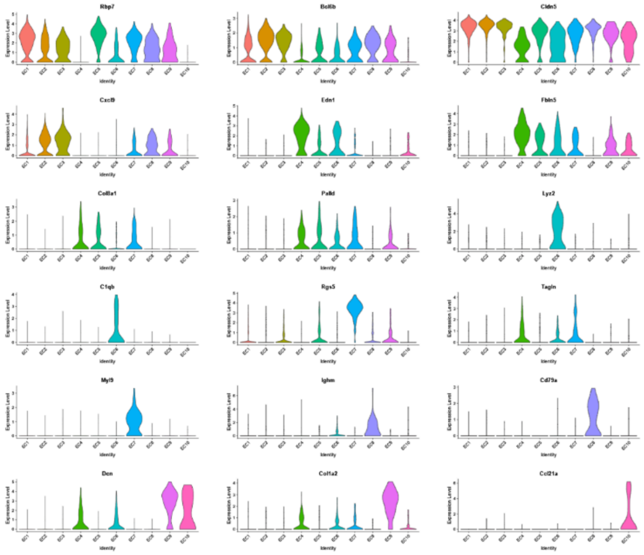

S2D

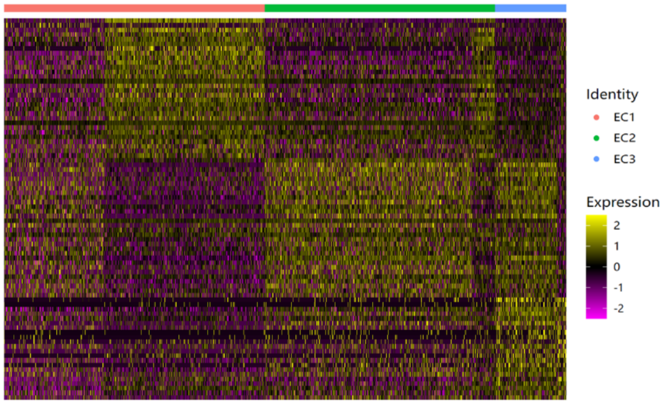

Supplementary Figure 2. (A) Constitution of total potential endothelial cells in pie chart. **(B)** Violin plots of upregulated genes of each cluster. **(C)** Violin plots demonstrate endothelial markers Nos3, Notch1 and Ptprb in the ten endothelial subpopulations. **(D)** Heatmap of upregulated genes compared between EC1, EC2 and EC3.

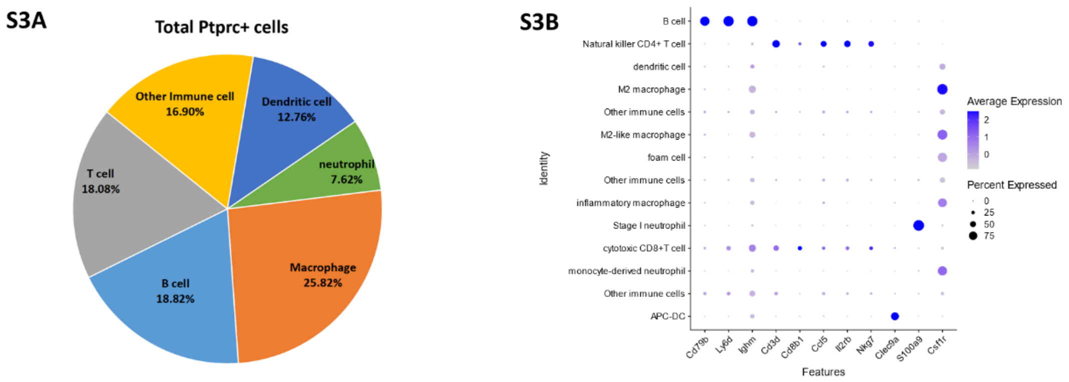

Supplementary Figure 3. (A) Pie chart of total Ptprc cells extracted from total cell repertoire of chow and western diet Apoe-/- mice. **(B)** Dot plot of markers of each cluster.

S4A

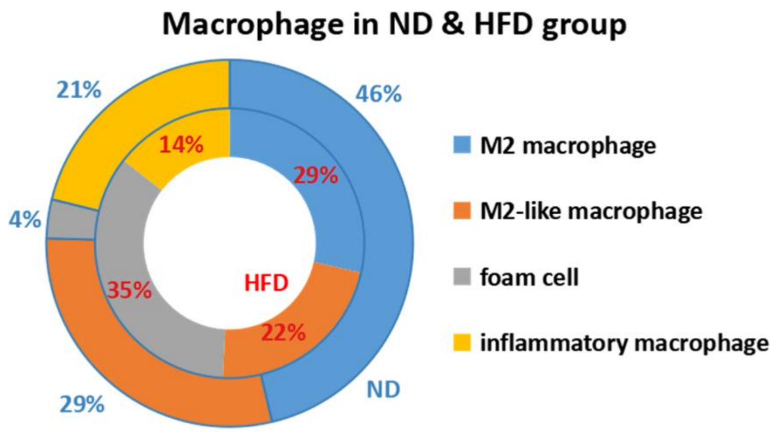

S4B

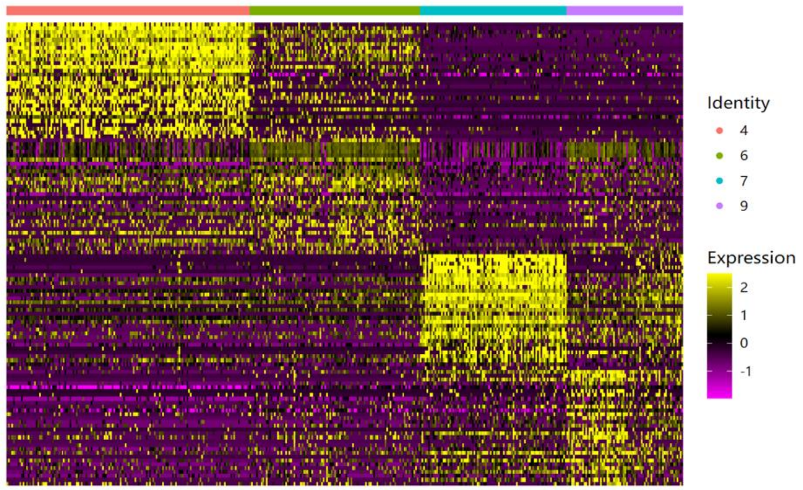

Supplementary Figure 4. (A) Proportion of each type of macrophage identified in the chow diet repertoire and western diet cell repertoire. **(B)** Heat map of top 30 upregulated genes of identified macrophages.

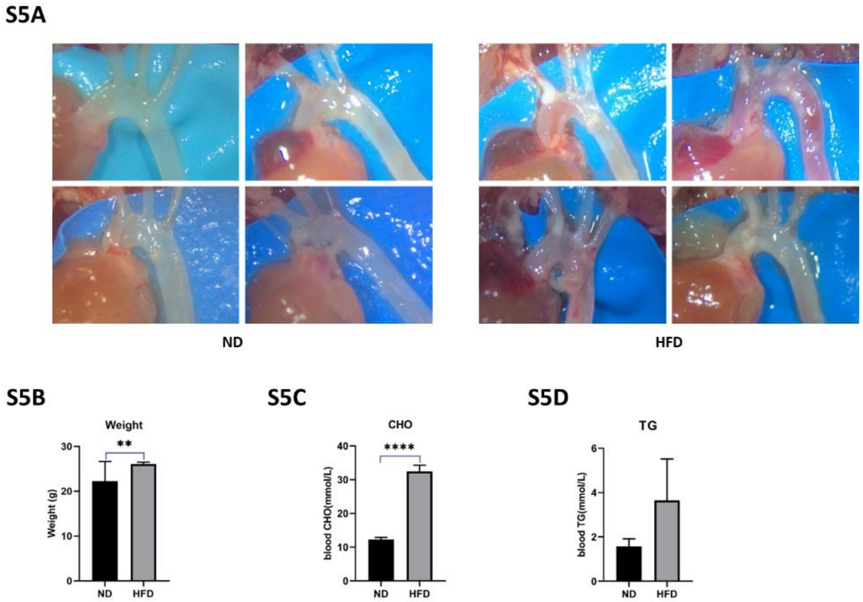

Supplementary Figure 5. Physical differences between chow and western diet group. **(A)** morphology of aorta under microscope. **(B)** Body weight of chow/western diet Apoe-/- mice. **(C)** Cholesterol level. **(D)** Triglyceride level in blood following 3 hours of fasting.
